# CRID3, a blocker of apoptosis associated speck like protein containing a card, ameliorates murine spinal cord injury by improving local immune microenvironment

**DOI:** 10.1186/s12974-020-01937-8

**Published:** 2020-08-29

**Authors:** Yu-Qing Chen, Sai-Nan Wang, Yu-Jiao Shi, Jing Chen, Shu-Qin Ding, Jie Tang, Lin Shen, Rui Wang, Hai Ding, Jian-Guo Hu, He-Zuo Lü

**Affiliations:** 1 Clinical Laboratory, The First Affiliated Hospital of Bengbu Medical College, 233004 Bengbu, Anhui People’s Republic of China; 2 Anhui Key Laboratory of Tissue Transplantation, The First Affiliated Hospital of Bengbu Medical College, 287 Chang Huai Road, Bengbu, 233004 Anhui People’s Republic of China; 3 Department of Immunology, Bengbu Medical College, 233030 Bengbu, Anhui People’s Republic of China; 4 Anhui Key Laboratory of Infection and Immunity, Bengbu Medical College, 233030 Bengbu, Anhui People’s Republic of China

**Keywords:** Spinal cord injury, CRID3, Inflammasome, Apoptosis-associated speck-like protein containing a card, Immune microenvironment, Locomotor recovery

## Abstract

**Background:**

After spinal cord injury (SCI), destructive immune cell subsets are dominant in the local microenvironment, which are the important mechanism of injury. Studies have shown that inflammasomes play an important role in the inflammation following SCI, and apoptosis-associated speck-like protein containing a card (ASC) is the adaptor protein shared by inflammasomes. Therefore, we speculated that inhibiting ASC may improve the local microenvironment of injured spinal cord. Here, CRID3, a blocker of ASC oligomerization, was used to study its effect on the local microenvironment and the possible role in neuroprotection following SCI.

**Methods:**

Murine SCI model was created using an Infinite Horizon impactor at T9 vertebral level with a force of 50 kdynes and CRID3 (50 mg/kg) was intraperitoneally injected following injury. ASC and its downstream molecules in inflammasome signaling pathway were measured by western blot. The immune cell subsets were detected by immunohistofluorescence (IHF) and flow cytometry (FCM). The spinal cord fibrosis area, neuron survival, myelin preservation, and functional recovery were assessed.

**Results:**

Following SCI, CRID3 administration inhibited inflammasome-related ASC and caspase-1, IL-1β, and IL-18 activation, which consequently suppressed M1 microglia, Th1 and Th1Th17 differentiation, and increased M2 microglia and Th2 differentiation. Accordingly, the improved histology and behavior have also been found.

**Conclusions:**

CRID3 may ameliorate murine SCI by inhibiting inflammasome activation, reducing proinflammatory factor production, restoring immune cell subset balance, and improving local immune microenvironment, and early administration may be a promising therapeutic strategy for SCI.

## Background

Spinal cord injury (SCI) is the damage of the spinal cord caused by various reasons, resulting in paralysis, loss of sensation, and related organ dysfunction [1]. SCI has a high disability rate and currently lacks effective treatment, so it is a serious disaster to the patients with SCI in physical, psychological, and economic aspects, and it is also one of the most serious public problems in the world [1, 2]. Therefore, exploring the effective treatment of SCI is of great significance for improving the quality of life of patients and reducing the burden of social medical care.

The pathological process of SCI includes primary injury and secondary injury. Primary injury refers to the direct injury of mechanical force to the spinal cord, which cannot be interfered clinically [3]. Secondary injury refers to local inflammation, edema, ischemia, and electrolyte changes, among which inflammation is one of the main damage factors [4, 5]. The inflammatory response is characterized by infiltration and activation of inflammatory cells in the injured spinal cords, which leads to the increase of inflammatory cells and inflammatory factors, and formation of inflammatory microenvironment, finally leading to spinal cord dysfunction [6]. Therefore, early and effective anti-inflammatory treatment and improvement of local immune microenvironment are important for protecting residual neurons and promoting functional recovery.

Inflammasome is a high molecular weight multi-protein complex discovered by Martinon et al. in 2002 [7]. It is mainly composed of three parts: intracellular pattern recognition receptor, caspase-1, and adaptor protein (apoptosis-associated speck-like protein containing a card, ASC). When encountering external or internal stimuli, inflammasome can recognize pathogen-associated molecular patterns (PAMPs) or damage-associated molecular patterns (DAMPs) from the host through pattern recognition receptors, and then ASC can recruit and activate pro-caspase-1 into caspase-1, which can further cleave and activate IL-1 family cytokines (such as IL-1β and IL-18), and finally lead to the downstream inflammatory cascade and pyroptosis [7–11].

In the central nervous system (CNS), inflammasomes are found in neurons, astrocytes, and microglia [12–15]. The intracellular pattern recognition receptors are NLRP1 and AIM2 in neurons, NLRP2 in astrocytes, and NLRP3 in microglia [12, 13]. NLRP1 inflammasome is composed of NLRP1, caspase-1, caspase-11, ASC, and X-linked inhibitor of apoptosis protein (XIAP), which was found by de Rivero Vaccari in the injured spinal cord in 2008 [14]. NLRP2 inflammasome has been found in the cultured astrocytes. It is composed of NLRP2, caspase-1, and ASC. It interacts with P2X7 receptor and pannexin-1, a channel protein, and can be activated by a high concentration of ATP [15]. NLRP3 complex is the best-characterized inflammasome, which contains NLRP3, caspase-1, and ASC. Recent researches also confirmed that it has obvious expression and activation following SCI [16, 17]. These demonstrated that inflammasomes may play an important role in the inflammation of SCI. Because ASC is the adaptor protein shared by inflammasomes, we speculated that ASC can be used as a target to inhibit the activation of inflammasomes, so as to improve the local immune microenvironment of SCI and reduce nerve damage. In this study, CRID3, a blocker of ASC oligomerization [18], was used to study its effect on the local microenvironment and the possible role in neuroprotection following SCI.

## Methods

### Animals

A total of 130 female C57BL/6 mice (weight, 18-20 g; age, 8 weeks old) obtained from Chang Zhou Cavens Laboratory Animal Ltd. Animals were housed in ventilated cages and maintained on a 12-h light/dark cycle with ad libitum access to food and water. Ambient room temperature was maintained at 20 ~ 22 °C with 30 ~ 70% humidity. Animal care following surgery complied with the regulations for the management of experimental animals (revised by the Ministry of Science and Technology of China in June 2004). The study was approved by the Institutional Committee on Animal Care, Use, and Research of the Bengbu Medical College. During the experiment, 10 mice died and 120 survived. Table 1 provided a comprehensive description of the total number of mice used per group in each experiment.

**Table 1:** The total number of mice used per group in each experiment

Description			Animal number
Mice	Experiment		
**Survived**	Western blot (WB)		18
	Immunohistofluorescence (IHF)		18
	Flow cytometry (FCM)	Microglia and macrophages, M1, and M2	18
		Th1, Th2, and Th17	18
		Treg and Tc	18
	Histology and behavior		30
**Died**	N/A		10
**Total**	N/A		130

### Contusive SCI and drug injection

An Infinite Horizon impactor (Precision Systems and Instrumentation) was used to perform contusive SCI, as previously described [19, 20]. The mice were first anesthetized with a cocktail of ketamine (80 mg/kg)/xylazine (10 mg/kg) intraperitoneally injection, and then the T9 lamina was excised. The spine was stabilized by clamping the T7 and T11 spinous processes, and then a moderate SCI model was created using a rod (1.3 mm in diameter) with a force of 50 kdynes. Sham-operated (sham) mice only received a laminectomy without contusive injury. Post-surgically, mice were placed in a temperature- and humidity-controlled chamber. Manual bladder emptying was performed three times daily until reflex bladder emptying was established. To relieve the postoperative pain, meloxicam (5 mg/kg, CSNpharm, IL, USA) were injected subcutaneously at 12 h time intervals following surgery for 7 days. To prevent infections, animals were daily provided with chloramphenicol (50 mg/kg) via drinking water. The spinal cord-injured mice were randomly assigned to the vehicle control or CRID3 injection groups. The CRID3 concentration chosen for this study was based on published reports [21–23]. Mice were intraperitoneally injected with 0.01 M PBS (pH 7.4) or CRID3 (Target Molecule Corp., 50 mg/kg prepared in PBS) immediately following injury one dose per day and continuing for 7 days.

### Western blot analysis

Three days following surgery, the mice were euthanized with a cocktail of ketamine (80 mg/kg)/xylazine (10 mg/kg) intraperitoneally injection and perfused with 10 ml cold 0.01 M PBS (pH 7.4). After perfusion, the 5-mm spinal cord segments containing the injury epicenter (or the same spinal cord segments in the sham group) were removed (*n* = 6 in every group). Total protein was extracted from the spinal cords and western blot analysis was performed as previously described [24]. The primary and secondary antibodies used were listed in Table 2. Finally, an ECL kit (Cat. # 35055, Pierce) was used to observe the immunoreactive protein. Gel-Pro analyzer (Media Cyrnistic, Silver Spring, MD) was used to digitize the membrane and determine the band’s density.

**Table 2:** Table of antibodies used

Antigen	Host species and clone	Cat. # or Lot #	RRID	Conjugation	Source	Used concentration	Methods
ASC	Rabbit polyclonal	abx013852			Abbexa	1:1000	WB
caspase-1	Rabbit monoclonal	ab179515			Abcam	1:1000	
β-actin	Rabbit polyclonal	BL005B			Biosharp	1:2000	
IL-18	Rabbit polyclonal	PA5-79481	AB_2746597		Invitrogen	1:1000	
IL-1β	Rabbit polyclonal	ab9722			Abcam	1:1000	
Rabbit IgG (H + L)	Goat polyclonal	BL003A		HRP	Biosharp	1:10,000	
CD11b	Rat monoclonal	14-0112-82	AB_467108		Invitrogen	1:200	IHF
CD45	Rat monoclonal	14-0451-82	AB_467251				
CD68	Rat monoclonal	MA5-16674	AB_2538168				
CCR7	Rabbit polyclonal	ab191575			Abcam		
Arg1	Rabbit polyclonal	PA5-29645	AB_2547120		Invitrogen		
CD4	Rat monoclonal	14-9766-82	AB_2573008				
CD4	Rabbit polyclonal	PA5-87425	AB_2804136				
GATA3	Rabbit polyclonal	PA5-20892	AB_11154392				
T-bet	Rabbit polyclonal	PA5-40573	AB_2576589				
ROR gamma (t)	Rabbit polyclonal	PA5-23148	AB_2540675				
FOXP3	Rat monoclonal	14-4776-82	AB_467554				
Rat IgG (H + L)	Goat polyclonal	112-095-143	AB_2338199	Fluorescein (FITC)	Jackson ImmunoResearch		
Rabbit IgG (H + L)	Goat polyclonal	111-025-144	AB_2337932	Rhodamine (TRITC)			
CCR7	Rat monoclonal	47-1971-82	AB_2573974	APC-eFluor 780 (AF780)	Invitrogen	0.25 μg/test	FCM
IgG2b kappa isotype control	Rat	47-4321-82	AB_1271997	APC-eFluor 780 (AF780)		0.25 μg/test	
CD11b	Rat monoclonal	12-0112-81	AB_465546	PE		0.125 μg/test	
CD3	Rat monoclonal	12-0032-82	AB_2811741	PE		0.25 μg/test	
IgG2b kappa isotype control	Rat	12-4031-82	AB_470042	PE		0.25 μg/test	
CD127	Rat monoclonal	48-1273-82	AB_2574039	eFlour 450		0.5 μg/test	
IgG2b kappa isotype control	Rat	48-4031-82	AB_1272017	eFlour 450		0.5 μg/test	
CD183	Armenian hamster monoclonal	62-1831-82	AB_2762747	Super Bright 436 (SB436)		0.5 μg/test	
IgG isotype control	Armenian Hamster	62-4888-82	AB_2717007	Super Bright 436 (SB436)		0.5 μg/test	
CD196	Rat monoclonal	50-7196-82	AB_11219682	eFlour 660		0.5 μg/test	
IgG2a kappa isotype control	Rat	50-4321-82	AB_10598503	eFlour 660		0.5 μg/test	
CD25	Rat monoclonal	47-0251-82	AB_1272179	APC-eFluor 780 (AF780)		0.5 μg/test	
IgG1 kappa isotype control	Rat	47-4301-80	AB_1271986	APC-eFluor 780 (AF780)		0.5 μg/test	
CD28	Syrian hamster monoclonal	45-0281-80	AB_925744	PerCP-Cyanine5.5 (PE-cy5.5)		0.5 μg/test	
IgG isotype control	Armenian hamster	45-4888-80	AB_906260	PerCP-Cyanine5.5 (PE-cy5.5)		0.5 μg/test	
CD3	Armenian hamster monoclonal	47-0031-82	AB_11149861	APC-eFluor 780 (AF780)		0.5 μg/test	
IgG isotype control	Armenian hamster	47-4888-80	AB_1271978	APC-eFluor 780 (AF780)		0.5 μg/test	
CD4	Rat monoclonal	11-0041-82	AB_464892	FITC		0.25 μg/test	
CD68	Rat monoclonal	MA5-16676	AB_2538170	FITC		0.25 μg/test	
IgG2b kappa isotype control	Rat	11-4031-82	AB_470004	FITC		0.25 μg/test	
CD45	Rat monoclonal	17-0451-82	AB_469392	APC		0.125 μg/test	
IgG2b kappa isotype control	Rat	17-4031-82	AB_470176	APC		0.125 μg/test	

### Immunohistofluorescence

Seven days following surgery, the mice were euthanized as described above, then transcardially exsanguinated with 10 ml 0.01 M PBS (pH 7.4) followed by fixation with 20 ml ice-cold 4% PFA in 0.01 M PBS (pH 7.4). After perfusion, the 5 mm spinal cord segments containing the injury epicenter (or the same spinal cord segments in sham group) were removed (*n* = 6 in every group), postfixed overnight in 4% paraformaldehyde (PFA) in 0.01 M PBS (pH 7.4), and transferred to 30% sucrose in 0.01 M PBS (pH 7.4) at 4 °C overnight. Then, the segments were placed in OCT compound embedding medium (Tissue-Tek, Miles, Elkart, IN) and 5 μm frozen sections were obtained longitudinally using a cryostat (Leica CM1900, Bannockburn, IL), followed by thaw-mounting on poly-L-lysine-coated slides. For immunohistochemical assay, the primary antibodies (Table 2) were incubated overnight at 4 °C. The following day, after being rinsed with 0.01 M PBS, the sections were incubated with FITC- or rhodamine-conjugated secondary antibodies (Table 2) at 37 °C for 1 h. Finally, the slides were washed, coverslipped with installation medium containing nuclear dye Hoechst 33342 (0.5 μm, sigma), and examined using a ZWISS Axio observation microscope. The spinal cord cross sections from six mice in every group were used for cell quantification. Five complete sections per animal were analyzed in a blinded fashion, and cell numbers were calculated as cells in a set of five slides from rostral to caudal containing the injury epicenter as previously described [25].

### Flow cytometry

Seven days following surgery, the mice were euthanized as described above, then the 5 mm spinal cord segments containing the injury epicenter (or the same spinal cord segments in the sham group) were removed (*n* = 6 in every group), and the spinal cords were removed by insufflation and dissociated by gently grinding the tissue into a single-cell suspension through a 45-μm nylon mesh with the plunger of a syringe. The cells were isolated by Percoll (Amersham Pharmacia Biotech, Piscataway, NJ, USA) gradient centrifugation as previously described [26]. Different panel of fluorescent-labeled antibodies (Table 2) were used to identify different immune cell subtypes. After incubation at 4 °C for 30 min, cells were washed three times with 0.01 M PBS (pH 7.4), fixed with 1% PFA and analyzed using a BD Accuri flow cytometer (Becton Dickinson, San Diego, CA). Isotype-matched antibodies (Table 2) were used to control for non-specific staining that was subtracted from specific staining results. A minimum of 100,000 events were collected and analyzed by the FlowJo 7.6.1 software (TreeStar Inc., Ashland OR).

### Histological analyses

Six weeks following surgery, the spinal cords in all groups were collected (*n* = 10 in every group), fixed, wrapped, and cut into 5-μm sections were obtained longitudinally using a cryostat (Leica CM1900, Bannockburn, IL), followed by thaw-mounting on poly-L-lysine-coated slides as described in “Immunohistochemical assay.” Then, three sets of slides (each set containing serial sections spaced 0.25 mm apart) were stained with hematoxylin-eosin (HE, Beyotime Biotechnology, Shanghai, China), Luxol fast blue (LFB, Sigma-Aldrich, St. Louis, MO, USA), and Nissl staining (Beyotime Biotechnology) according to the manufacturer’s instructions, respectively, to identify fibrosis area, myelinated white matter, and residual ventral horn motoneurons. Lesion epicenter was defined as the section containing the least amount of spared white matter. The images of transverse sections were collected at the lesion epicenter and 0.25, 0.5, 1, and 1.5 mm rostral and caudal to the epicenter using a ZWISS Axio observation microscope. The myelinated white matter was quantified by Image pro-plus 5.1 (Media Cybernetics, Inc., Atlanta, GA, USA) and the number of surviving ventral horn neurons was confirmed by the exhibition of Nissl substance, euchromatic nucleus, and nucleolus [27]. The fibrosis area and cell quantification were performed using the ImageJ software (http://rsb.info.nih.gov/ij/; National Institutes of Health, Maryland, USA) in an unbiased stereological manner as previously described [28].

### Basso mouse scale for locomotion

Behavioral assessment was performed using BMS, a 10-point scale (0-9) based on observations of hind-limb movements of mouse freely moving in an open field [29]. The BMS scores were evaluated at 1 and 3 days, then 1, 2, 3, 4, 5-, and 6-weeks following injury. During the evaluation, mice were walked freely on the open-field surface for 4 min, while being observed by two blinded scorers.

### Statistical analyses

The histological and behavioral data were analyzed using repeated measures, two-way ANOVA statistical test, followed by post hoc analysis for multiple comparisons. Other data were analyzed using non-parametric Kruskal-Wallis ANOVA, followed by individual Mann-Whitney *U* tests. Statistical differences were considered significant at *P* < 0.05.

## Results

### CRID3 inhibits ASC-related molecule expression and action following SCI

**Fig. 1:**
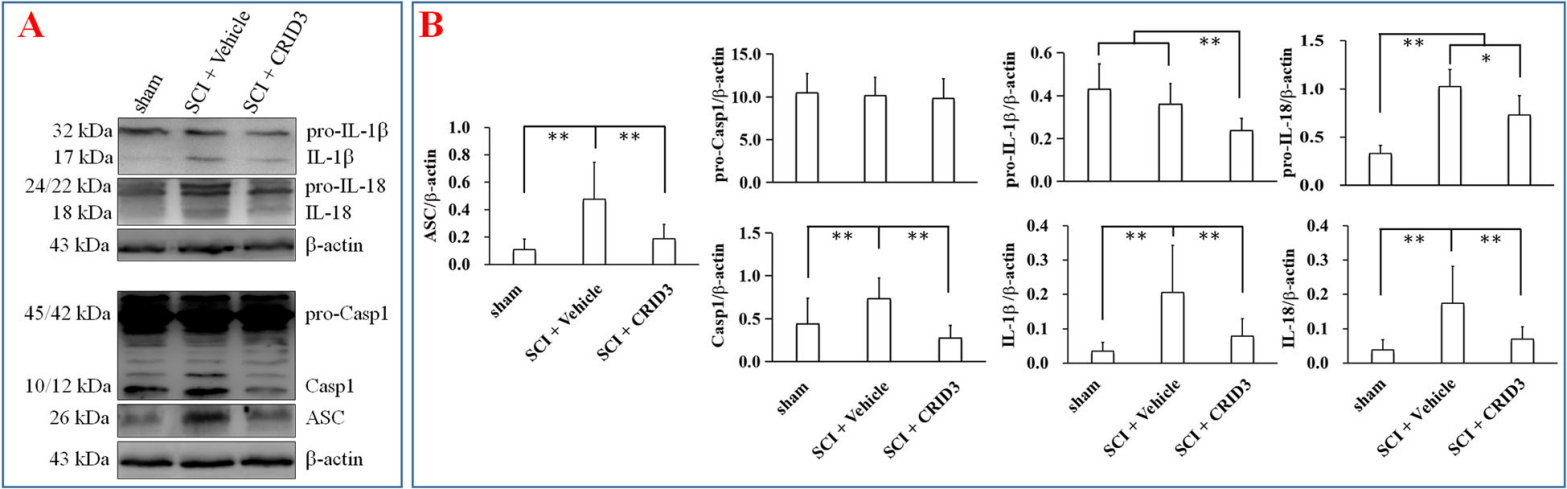
CRID3 inhibits ASC-related molecule expression and action following SCI. **a** The representative WB results of the expression of ASC (26 kDa), pro-caspase-1 (45/42 kDa), cleaved-caspase-1 (12/10 kDa), pro-IL-1β (32 kDa), IL-1β (17 kDa), pro-IL-18 (24/22 kDa), IL-18 (18 kDa), and β-actin (43 kDa). **b** The statistical graphs. Data represent the mean ± SD (*n* = 6). *P < 0.05, **P < 0.01 (non-parametric Kruskal-Wallis ANOVA, followed by individual Mann-Whitney *U* tests)

To verify the effect of CRID3 on ASC-related molecule expression and activation, the homogenate extracts obtained from sham, vehicle, and CRID3-treated injured spinal cords were detected by WB. As shown in Fig. 1, ASC (26 kDa), cleaved-caspase-1 (12/10 kDa), IL-1β (17 kDa), pro-IL-18 (24/22 kDa), and IL-18 (18 kDa) significantly increased in SCI (vehicle) compared with sham group (all *P* < 0.01, *n* = 6), while they significantly decreased in SCI (CRID3) compared with SCI (vehicle) group (*P* < 0.05 or 0.01, *n* = 6). Pro-caspase-1 (45/42 kDa) had no differences among the three groups (all *P* > 0.05, *n* = 6). Although the 32 kDa pro-IL-1β had no difference between the sham and SCI (vehicle) group (*P* > 0.05, *n* = 6), it significantly decreased in SCI (CRID3) compared with SCI (vehicle) group (*P* < 0.01, *n* = 6). These are enough to indicate that CRID3 can inhibit ASC-related molecule expression and activation following SCI.

### CRID3 inhibits SCI-induced microglia differentiation into M1 and increases them into M2 cells

**Fig. 2:**
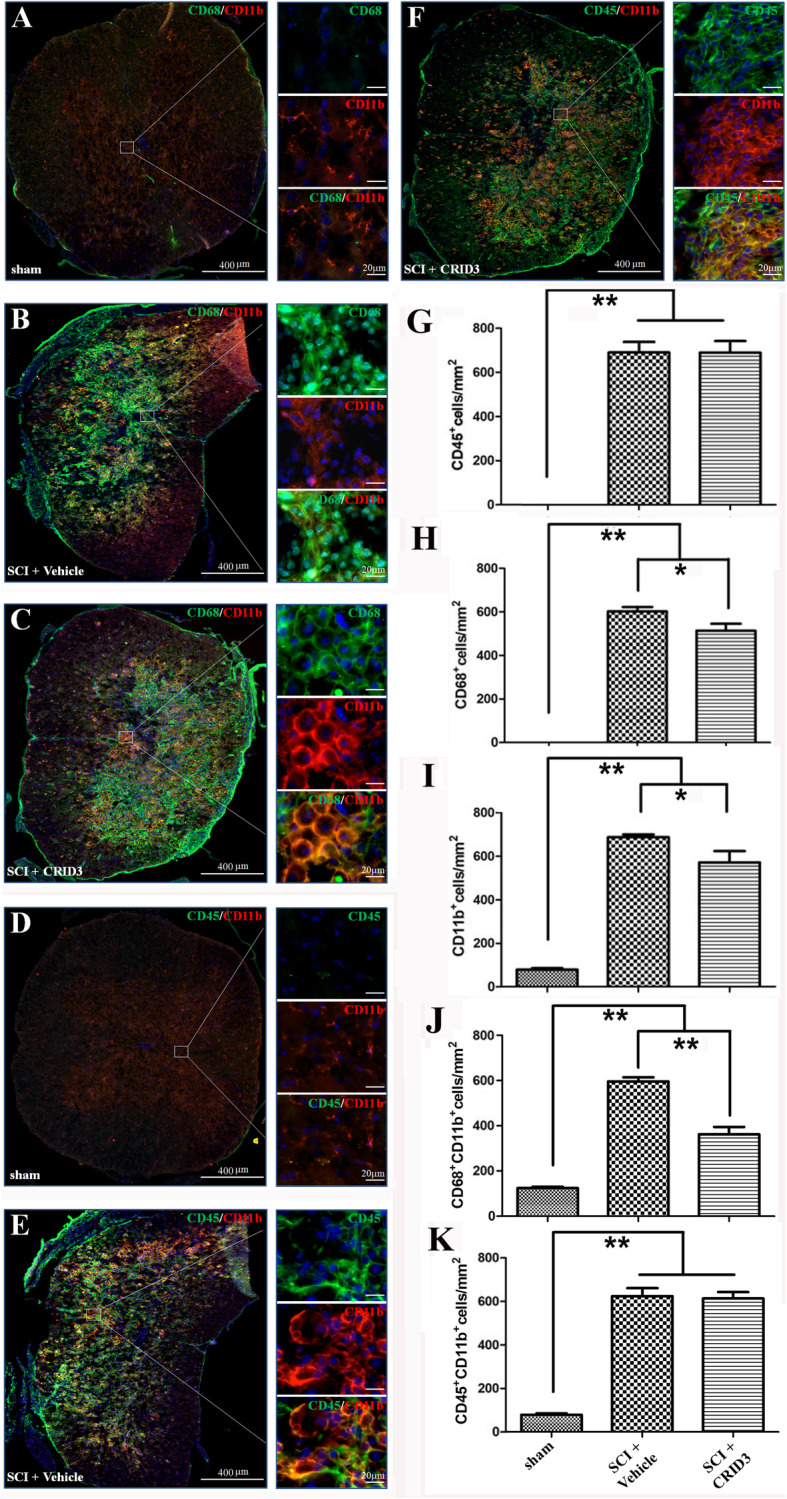
Effect of CRID3 on the number and status of microglia and infiltrated macrophages in the injured spinal cord as distinguished by IHF. (**a**-**f**) Representative images of CD11b (red) and CD68 (green) (**a**-**c**) or CD45 (**e**-**f**) expression in the spinal cords in sham, SCI (vehicle), and SCI (CRID3) groups. Cells were counterstained with Hoechst 33342 (blue) to visualize nuclei. (**g**-**k**) Quantitative analysis of CD45^+^ (**g**), CD68^+^ (**h**), CD11b^+^ (**i**), CD68^+^CD11b^+^ (**j**), and CD45^+^CD11b^+^ (**k**) cells in the indicated groups. Data represent the mean ± SD (*n* = 6). *P < 0.05, **P < 0.01 (non-parametric Kruskal-Wallis ANOVA, followed by individual Mann-Whitney *U* tests)

To determine the effect of CRID3 on the number and status of microglia and infiltrated macrophages in the injured spinal cord, we used immunofluorescence double staining to label CD11b with CD68 and CD45, respectively. CD45 is the common marker of peripheral leukocytes [30, 31], CD11b is the common marker of macrophages and microglia [32], CD68 is the common marker of activated macrophages and microglia [33, 34]. CD45^+^CD11b^+^ can accurately label macrophages from peripheral blood, and CD68^+^CD11b^+^ can accurately label activated macrophages and microglia. As shown in Fig. 2, CD68^+^ cells (a) and CD45^+^ cells (d) in sham group were very rare, they significantly increased following SCI (b and e) compared with sham group (g and h, *P* < 0.01, *n* = 6). After administration of CRID3 (c and f), CD45^+^ cells in SCI (CRID3) group had no significant difference compared with SCI (vehicle) group (g, *P* > 0.05, *n* = 6), while CD68^+^ cells decreased (c), which was significantly different from SCI (vehicle) group (h, *P* < 0.05, *n* = 6). In sham group (a and d), typical microglia with a number of tiny processes could be detected. After SCI, their morphology became round or oval (b and e), and the number increased significantly (i). There was a significant difference between SCI (vehicle) and sham groups (i, *P* < 0.05, *n* = 6). After CRID3 administration, CD11b^+^ cells decreased (c, f, and i), which was significantly different from SCI (vehicle) group (i, *P* < 0.01, *n* = 6). CD68^+^CD11b^+^ cells (a) and CD45^+^CD11b^+^ cells (d) were not detected in the sham group, and both increased significantly following SCI (b, e, j, and k, both *P* < 0.01, *n* = 6). After CRID3 administration, CD68^+^CD11b^+^ cells were significantly decreased (C) compared with SCI (vehicle) group (j, *P* < 0.01, *n* = 6), however, CD45^+^CD11b^+^ cells (f) had no significant difference between SCI (vehicle) and SCI (CRID3) groups (k, *P* > 0.05, *n* = 6). These results indicate that following SCI, the infiltration of peripheral leukocytes could be detected in the injured spinal cords, among them macrophages are dominant, and the activation of macrophages and/or local microglia could also be detected; CRID3 has no significant effect on the infiltration of macrophages, but it can inhibit the activation of macrophages and/or local microglia.

**Fig. 3:**
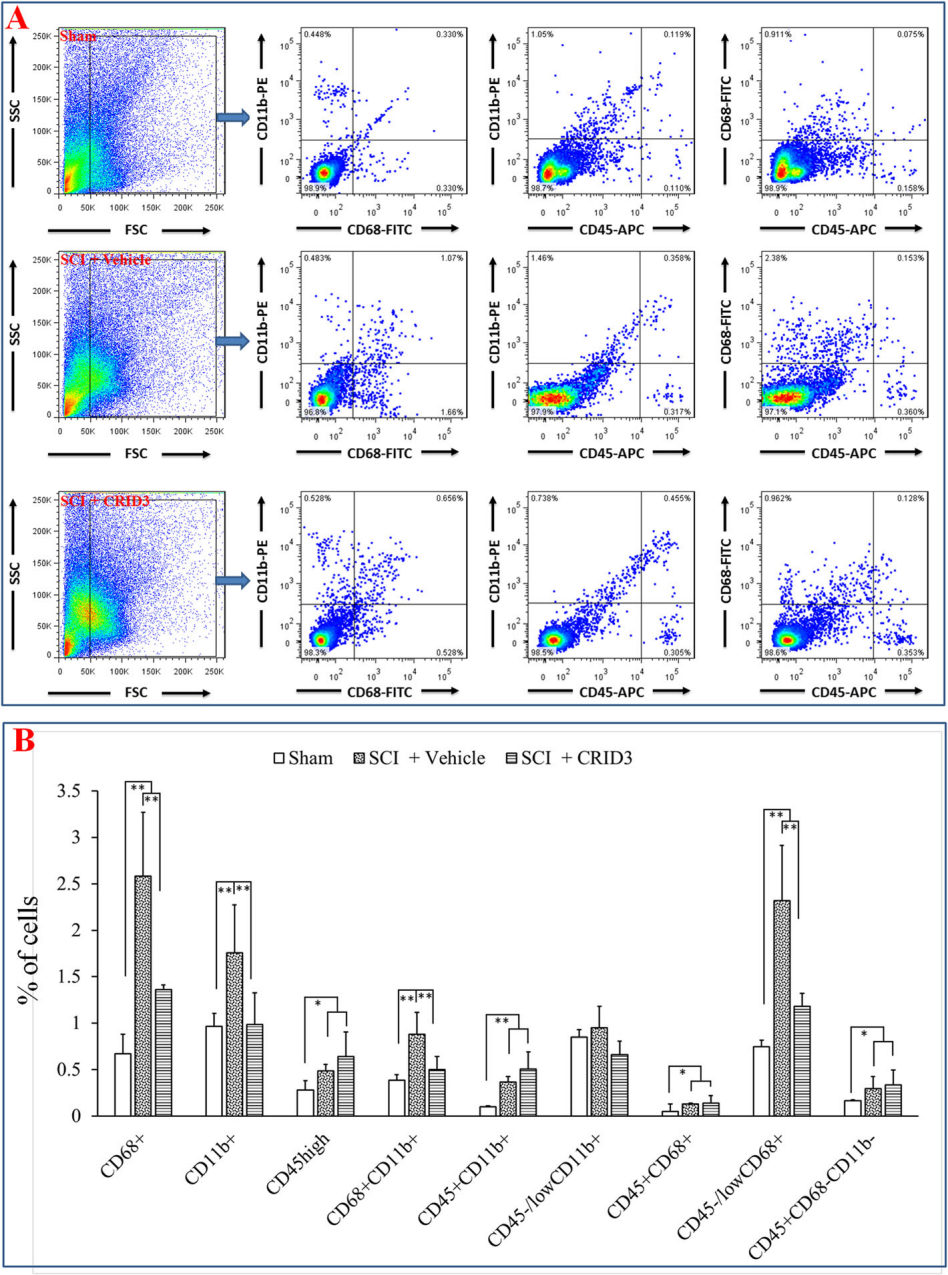
Effect of CRID3 on the number and status of microglia and infiltrated macrophages in the injured spinal cord as distinguished by FCM. **a** Representative images of FCM in the spinal cords in sham, SCI (vehicle), and SCI (CRID3) groups. In the FSC/SSC pseudocolor plot, the same size “region” of the nucleated cells was set for each sample, and then analyze the proportion of each cell population. **b** Quantitative analysis of the indicated cells in the indicated groups. Data represent the mean ± SD (*n* = 6). *P < 0.05, **P < 0.01 (non-parametric Kruskal-Wallis ANOVA, followed by individual Mann-Whitney *U* tests)

To determine the effect of CRID3 on the proportion and status of microglia and infiltrated macrophages in the injured spinal cord, the panel of CD45, CD11b, and CD68 molecules were detected by FCM. Because of there might be low expression of CD45 in the activated microglia [35, 36], we defined CD45^high^ cells as peripheral infiltrated leukocytes, CD68^+^CD11b^+^ cells as activated macrophages and microglia, CD45^high^CD11b^+^ cells as peripheral derived macrophages, CD45^−/low^CD11b^+^ cells as microglia, CD45^high^CD68^+^ cells as activated peripheral derived macrophages, CD45^−/low^CD68^+^ cells as activated microglia, and CD45^high^CD68^−^CD11b^−^ cells as peripheral-derived leukocytes excluding macrophages. As shown in Fig. 3, in the FSC/SSC pseudocolor plot, we set the same size “region” of the nucleated cells for each sample, and then analyze the proportion of each cell population. The results showed that except CD45^−/low^CD11b^+^ cells, the proportions of the other cell subsets in the SCI (vehicle) group were significantly increased compared with the sham group (*P* < 0.01 or 0.05, *n* = 6), and CRID3 could significantly decreased the cell subsets of CD68^+^, CD11b^+^, CD68^+^CD11b^+^, and CD45^−/low^CD68^+^ (all *P* < 0.01, *n* = 6) and had no influence on the other cell subsets (all *P* > 0.05, *n* = 6).

The results of IHF and FCM showed that the microglia in the sham-opened spinal cords are inherent and the infiltrated macrophages are very rare. Following SCI, the number of infiltrated macrophages and local microglia increases significantly and the state is activated, and the local-activated microglia are dominant. After CRID3 treatment, the number and proportion of activated microglia decreases significantly.

**Fig. 4:**
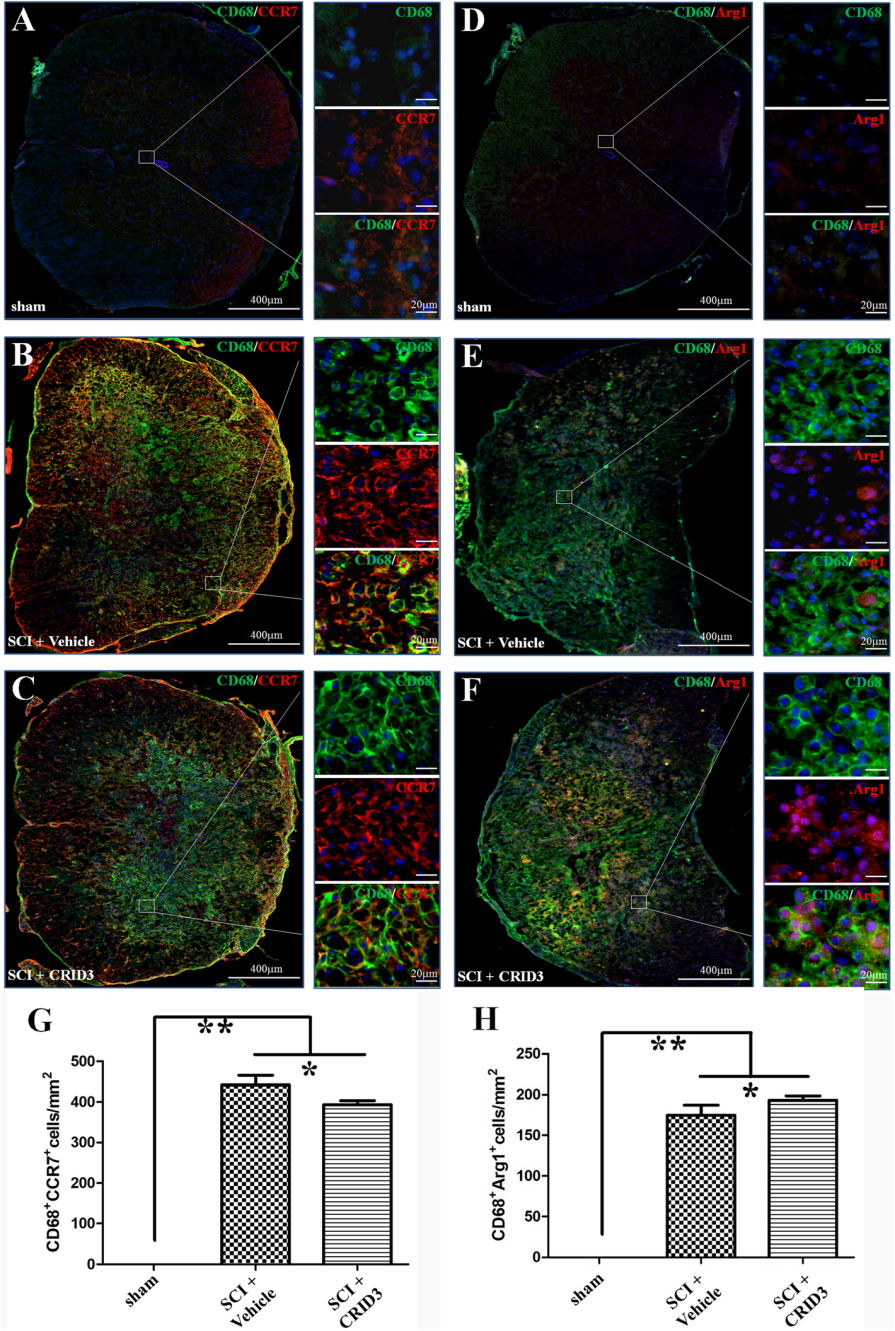
Effect of CRID3 on M1 and M2 cells in the injured spinal cord as distinguished by IHF. (**a**-**f**) Representative images of CD68 (green) and CCR7 (red) (**a**-**c**) or Arg1 (**e**-**f**) expression in the spinal cords in sham, SCI (vehicle), and SCI (CRID3) groups. Cells were counterstained with Hoechst 33342 (blue) to visualize nuclei. (**g** and **h**) Quantitative analysis of CD68^+^CCR7^+^ (**g**) and CD68^+^Arg1^+^ (**h**) cells in the indicated groups. Data represent the mean ± SD (*n* = 6). *P < 0.05, **P < 0.01 (non-parametric Kruskal-Wallis ANOVA, followed by individual Mann-Whitney *U* tests)

To further clarify the effect of CRID3 on M1 and M2 cell subsets, we continued the strategy of combination of IHF and FCM. When IHF was used to distinguish M1 and M2 cell subsets, CD68, a common marker of activated macrophages and/or microglia, as well as M1- (CCR7) and M2- (Arg1) specific markers [33] were detected. As shown in Fig. 4, CD68^+^CCR7^+^ cells are M1 cells (a-c), CD68^+^Arg1^+^ cells are M2 cells (d-f). CD68^+^CCR7^+^ cells were very rare in sham group (a and g), increased significantly in SCI (vehicle) group (b and g, *P* < 0.01, *n* = 6), and decreased significantly in SCI (CRID3) group (c and g, *P* < 0.05, *n* = 6). CD68^+^Arg1^+^ cells were also very rare in the sham group (d and h), increased significantly in SCI (vehicle) group (e and h, *P* < 0.01, *n* = 6), and further increased significantly in SCI (CRID3) group (f and h, *P* < 0.05, *n* = 6).

**Fig. 5:**
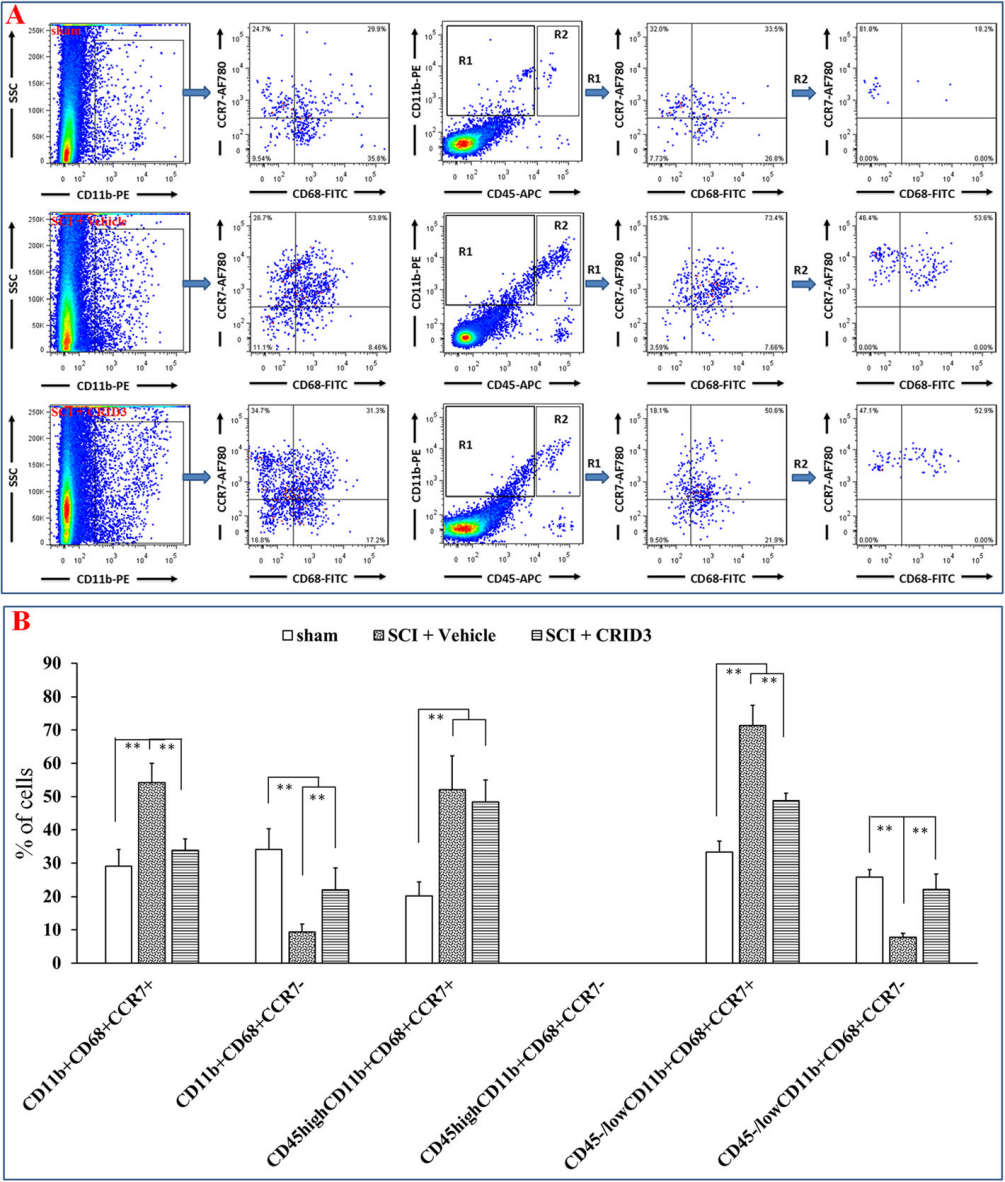
Effect of CRID3 on M1 and M2 cells in the injured spinal cord as distinguished by FCM. **a** Representative images of FCM in the spinal cords in sham, SCI (vehicle), and SCI (CRID3) groups. In the CD11b/SSC pseudocolor plots, the same size “region” of the nucleated CD11b^+^ cells was set for each sample, and then analyzed the proportion of each cell population in CD68/CCR7 and CD45/CD11b pseudocolor plots, respectively. In the CD45/CD11b pseudocolor plots, the “regions” of the CD11b^+^CD45^−/low^ cells (R1) and CD11b^+^CD45^high^ cells (R2) for each sample were further analyzed for the proportion of each cell population in CD68/CCR7. **b** Quantitative analysis of the indicated cells in the indicated groups. Data represent the mean ± SD (*n* = 6). **P < 0.01 (non-parametric Kruskal-Wallis ANOVA, followed by individual Mann-Whitney *U* tests)

When FCM was used to analyze M1 and M2 cells, we used a panel of CD68, CD45, CD11b, and CCR7 [31–33]. We defined CD11b^+^CD68^+^CCR7^+^ cells as M1, CD11b^+^CD68^+^CCR7^−^ cells as M2, CD45^high^CD11b^+^CD68^+^CCR7^+^ cells as peripheral-infiltrating M1, CD45^high^CD11b^+^CD68^+^CCR7^−^ as peripheral-infiltrating M2, CD45^−/low^CD11b^+^CD68^+^CCR7^+^ cells as microglia-derived M1, CD45^−/low^CD11b^+^CD68^+^CCR7^−^ cells as microglia-derived M2. As shown in Fig. 5a, in the CD11b/SSC pseudocolor plots, we set the same size “region” of the nucleated CD11b^+^ cells for each sample, and then analyzed the proportion of each cell population in CD68/CCR7 and CD45/CD11b pseudocolor plots, respectively. In the CD45/CD11b pseudocolor plots, the “regions” of the CD11b^+^CD45^−/low^ cells (R1) and CD11b^+^CD45^high^ cells (R2) for each sample were further analyzed for the proportion of each cell population in CD68/CCR7. The statistical results of Fig. 5b showed that the proportions of CD11b^+^CD68^+^CCR7^+^, CD45^high^CD11b^+^CD68^+^CCR7^+^, and CD45^−/low^CD11b^+^CD68^+^CCR7^+^ cells in SCI (vehicle) group were significantly increased compared with sham group (all *P* < 0.01, *n* = 6). CRID3 could significantly inhibit the proportions of CD11b^+^CD68^+^CCR7^+^ and CD45^−/low^CD11b^+^CD68^+^CCR7^+^ cells (both *P* < 0.01, *n* = 6); however, it has no influence on CD45^high^CD11b^+^CD68^+^CCR7^+^ cells (*P* > 0.05, *n* = 6). The proportions of CD11b^+^CD68^+^CCR7^−^ and CD45^−/low^CD11b^+^CD68^+^CCR7^−^ cells in the two SCI groups were significantly decreased compared with the sham group (both *P* < 0.01, *n* = 6). However, their proportions significantly increased following CRID3 treatment (both *P* < 0.01, *n* = 6). CD45^high^CD11b^+^CD68^+^CCR7^−^ cells were not detected in all groups.

The results of IHF and FCM suggest that there are few macrophages infiltrating into the normal spinal cord, the local microglia have both M1 and M2 types, and the proportion is balanced. After SCI, the infiltrated macrophages increase and mainly belong to M1 type, and most of local microglia transform into M1 type. CRID3 has no significant influence on the number and differentiation of infiltrated macrophages. However, as for the phenotype transformation of local microglia following SCI, CRID3 can not only significantly inhibit microglia transform into M1 but also promote them transform into M2 cells. Here, it should be emphasized that M1 cells derived from microglia might play more important roles than those derived from macrophages in the secondary inflammatory response following SCI.

### CRID3 inhibits SCI-induced Th1 and Th1Th17 differentiation and promotes Th2 differentiation

**Fig. 6:**
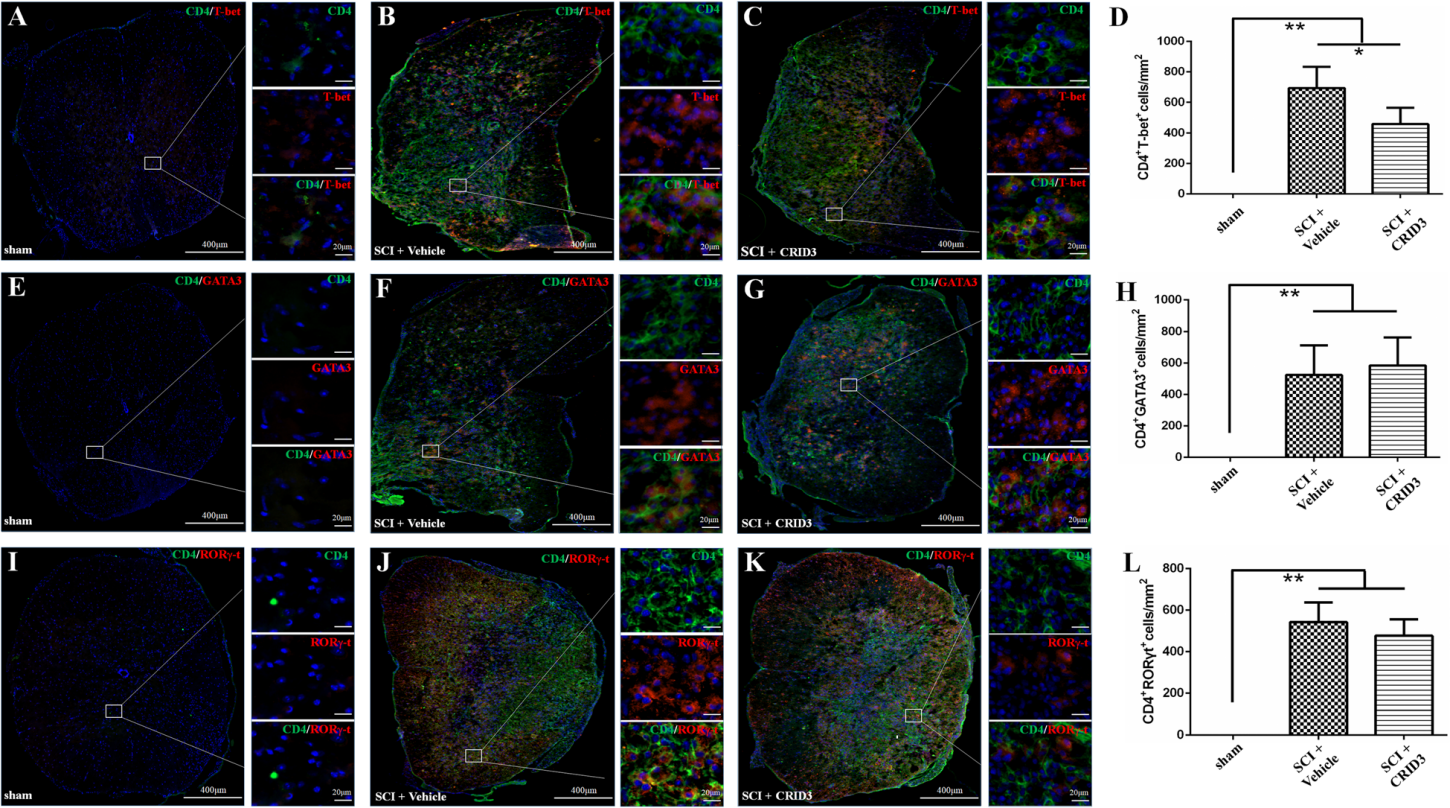
Effect of CRID3 on Th1, Th2, and Th17 cells in the injured spinal cord as distinguished by IHF. (**a**-**f**) Representative images of CD4 (green) and T-bet (red) (**a**-**c**) or GATA3 (red) (**e**-**f**) or (RORγ-t) (red) (**i**-**k**) expression in the spinal cords in sham, SCI (vehicle), and SCI (CRID3) groups. Cells were counterstained with Hoechst 33342 (blue) to visualize nuclei. (**d**, **h**, and **l**) Quantitative analysis of CD4^+^T-bet^+^ (**d**), CD4^+^GATA3^+^ (**h**), and CD4^+^RORγ-t^+^ (**l**) cells in the indicated groups. Data represent the mean ± SD (*n* = 6). *P < 0.05, **P < 0.01 (non-parametric Kruskal-Wallis ANOVA, followed by individual Mann-Whitney *U* tests)

To further clarify the effect of CRID3 on Th cell subsets, we continue the strategy of combination of IHF and FCM. When IHF was used to distinguish among different Th subsets, a general marker (CD4) [37], as well as Th1- (T-bet), Th2- (GATA3), and Th17- (RORγ-t) [38, 39] were detected. As shown in Fig. 6, CD4^+^T-bet^+^ cells are Th1 cells (a-c), CD4^+^GATA3^+^ cells are Th2 cells (e-g), CD4^+^RORγ-t^+^ cells are Th17 cells (i-k). All of these cells were very rare in the sham group (a, e, and i), increased significantly in SCI (vehicle) group (b, d, f, h, j, and l, all *P* < 0.01, *n* = 6). In SCI (CRID3) group, CD4^+^T-bet^+^ cells decreased significantly (c and d, *P* < 0.05, *n* = 6). However, the numbers of CD4^+^GATA3^+^ and CD4^+^ RORγ-t^+^ cells in SCI (vehicle) and SCI (CRID3) groups had no significant differences (g, h, k, and l, both *P* > 0.05, *n* = 6).

**Fig. 7:**
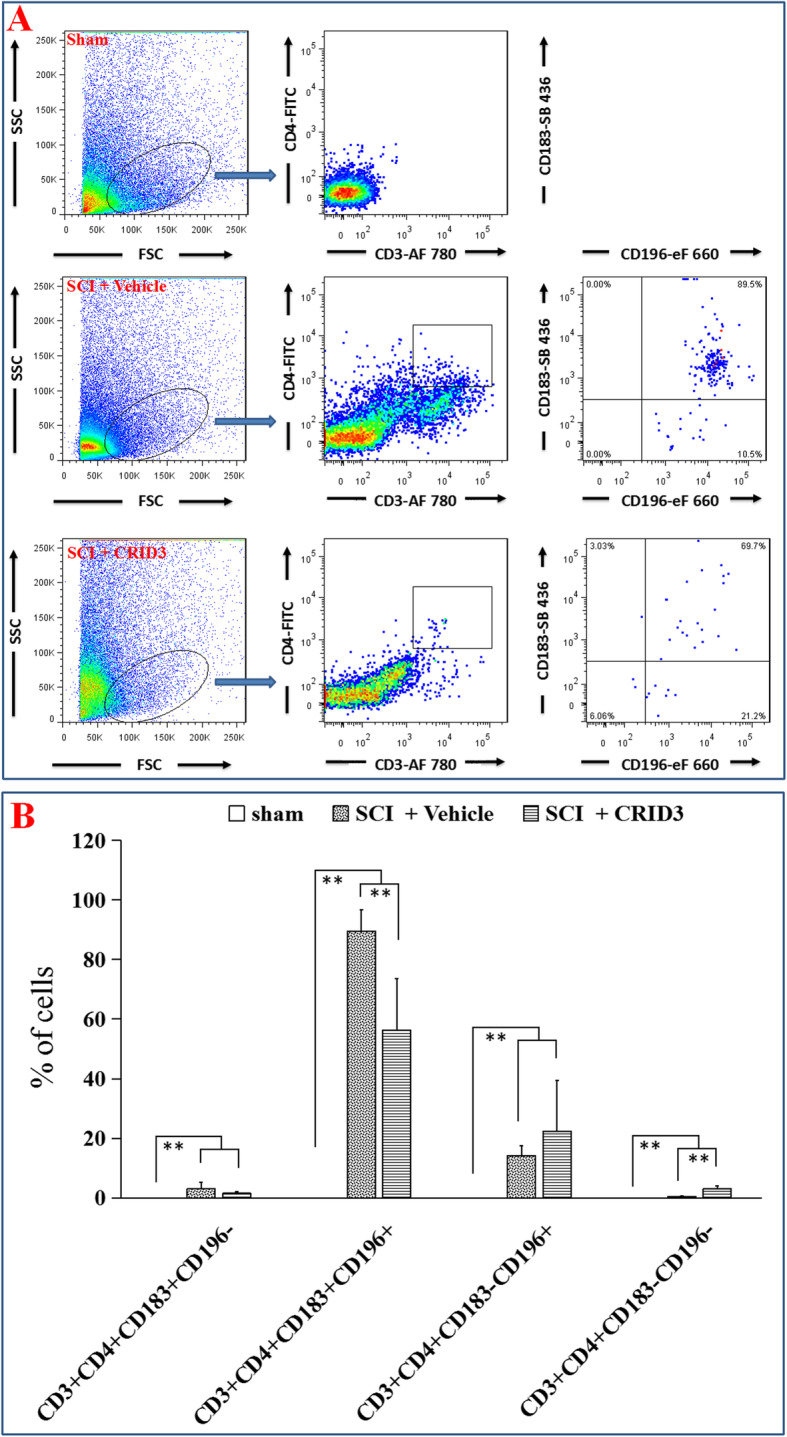
Effect of CRID3 on Th1, Th2, and Th17 cells in the injured spinal cord as distinguished by FCM. **a** Representative images of FCM in the spinal cords in sham, SCI (vehicle), and SCI (CRID3) groups. In the FSC/SSC pseudocolor plot, the same size “region” of lymphocytes was set for each sample, and then analyzed the proportion of each Th subsets in the “region” of CD3^+^CD4^+^ in CD3/CD4 pseudocolor plots. **b** Quantitative analysis of the indicated cells in the indicated groups. Data represent the mean ± SD (*n* = 6). **P < 0.01 (non-parametric Kruskal-Wallis ANOVA, followed by individual Mann-Whitney *U* tests)

When FCM was used to analyze Th cell subsets in the injured spinal cords, we used a panel of CD3, CD4, CD183, and CD196 [40–44]. Based on the previous reports, we defined CD3^+^CD4^+^CD183^+^CD196^−^ cells as Th1, CD3^+^CD4^+^CD183^+^CD196^+^ cells as Th1Th17, CD3^+^CD4^+^CD183^−^CD196^+^ cells as Th17, and CD3^+^CD4^+^CD183^−^CD196^−^ cells as Th2. As shown in Fig. 7a, in the FSC/SSC pseudocolor plot, we set the same size “region” of lymphocytes for each sample, and then analyzed the proportion of each Th subsets in the “region” of CD3^+^CD4^+^ in CD3/CD4 pseudocolor plots. The statistical results of Fig. 7b showed that the proportions of all Th subsets in SCI (vehicle) and SCI (CRID3) groups were significantly increased compared with the sham group (all *P* < 0.01, *n* = 6). However, CD3^+^CD4^+^CD183^+^CD196^+^ cells were dominant in all Th subsets, and its proportion in SCI (CRID3) group was significantly decreased compared with SCI (vehicle) group (*P* < 0.01, *n* = 6). Although few in number, the proportion of CD3^+^CD4^+^CD183^−^CD196^−^ cells in SCI (CRID3) group was significantly increased compared with SCI (vehicle) group (*P* < 0.01, *n* = 6). The other two subsets had no differences between the latter two groups (*P* > 0.05, *n* = 6), and their numbers were also very few. In addition, the numbers and proportions of regulatory T cell (Treg) and cytotoxic T cells (Tc) have also been detected. Although they both increase significantly after SCI, CRID3 has no significant effect on their numbers and proportions (Fig. S1 and S2, both *P* > 0.05, *n* = 6).

Combined with the results of IHF and FCM, it can be found that the number of Th1, Th2, and Th17 cells increases significantly after SCI, but in proportion, Th1Th17 cell subset is dominant. CRID3 can not only reduce Th1 cell number and Th1Th17 subset proportion but also increase Th2 cell proportion. The final results should be significant inhibition of Th1 and Th1Th17 differentiation and promotion of Th2 differentiation.

### CRID3 reduces fibrosis, increases myelination and residual neurons, and promotes functional recovery

**Fig. 8:**
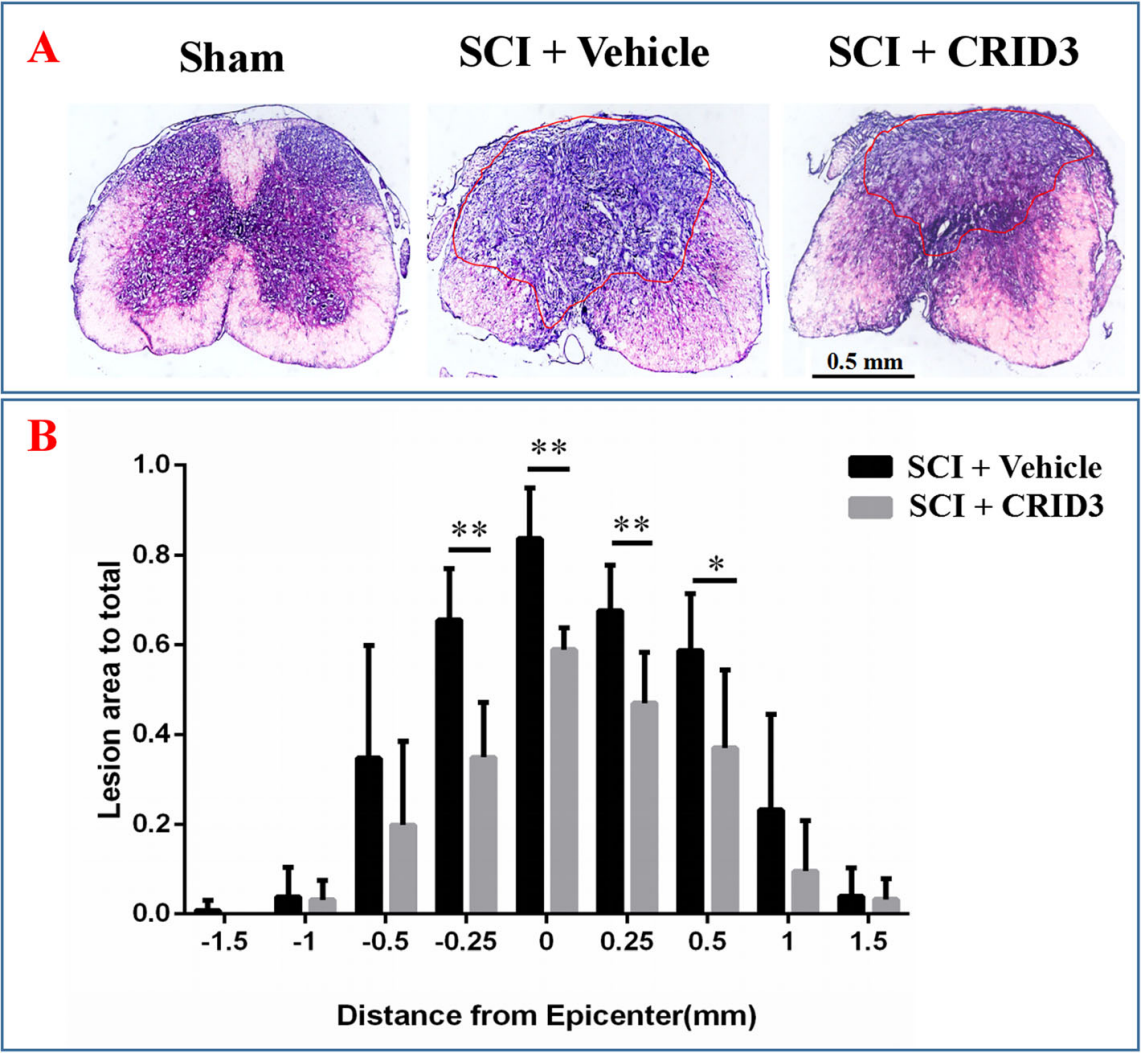
Quantitative analysis of the effect of CRID3 on fibrosis area 6 weeks after SCI. **a** HE-stained spinal cord cross section from the injury epicenter and the corresponding sections of sham-opened spinal cord. **b** Quantitative analysis of fibrosis area in different groups at various distances from the injury epicenter (0) and 0.25, 0.5, 1, and 1.5 mm rostral (+) and caudal (−) to the epicenter. Data represent the mean ± SD (*n* = 10). *P < 0.05, **P < 0.01 (repeated measures two-way ANOVA, followed by post hoc analysis for multiple comparisons)

**Fig. 9:**
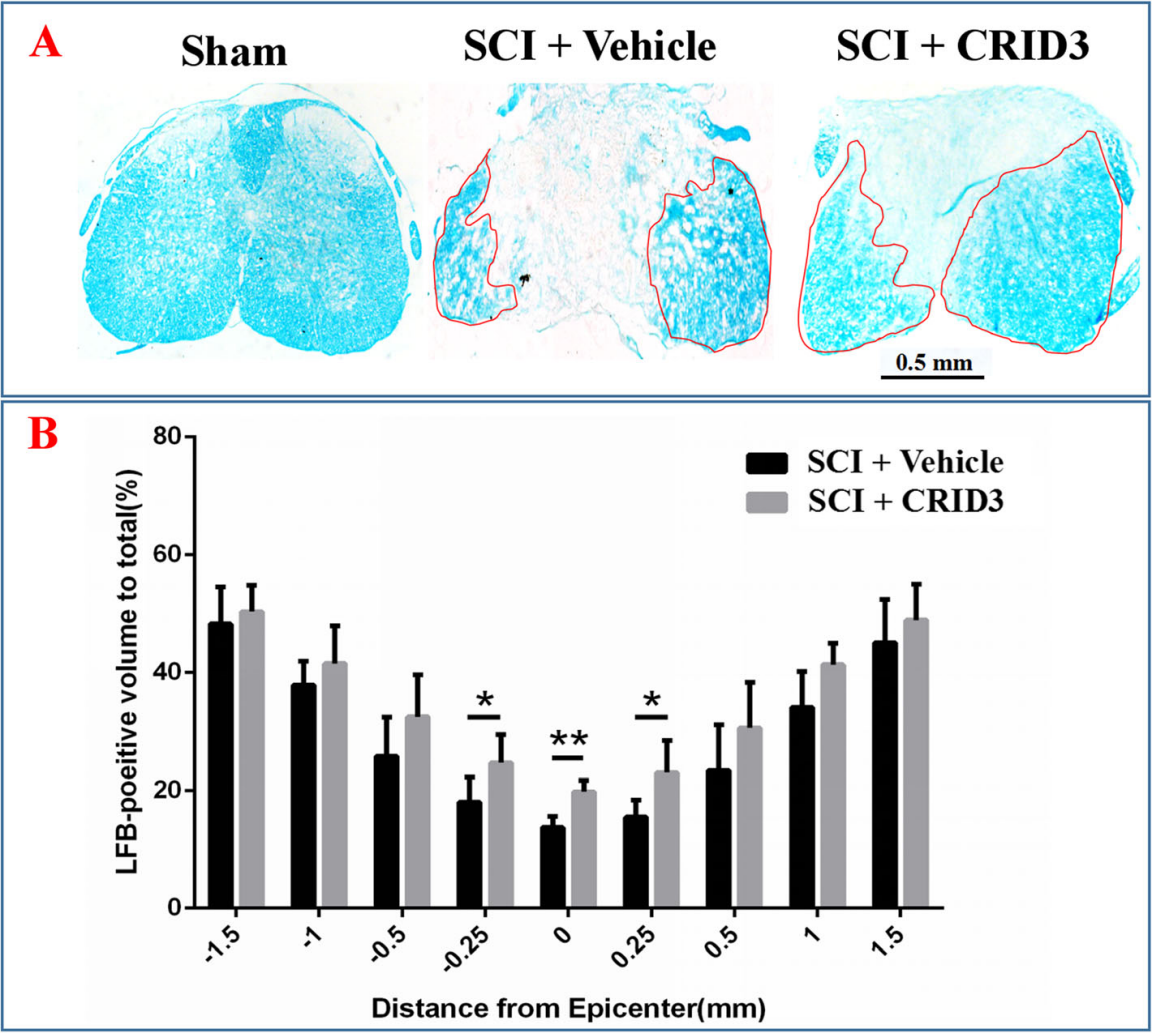
Quantitative analysis of the effect of CRID3 on residual myelination 6 weeks after SCI. **a** LFB-stained spinal cord cross section from the injury epicenter and the corresponding sections of sham-opened spinal cord. **b** Quantitative analysis of residual myelination in different groups at various distances from the injury epicenter (0) and 0.25, 0.5, 1, and 1.5 mm rostral (+) and caudal (−) to the epicenter. Data represent the mean ± SD (*n* = 10). *P < 0.05, **P < 0.01 (repeated measures two-way ANOVA, followed by post hoc analysis for multiple comparisons)

**Fig. 10:**
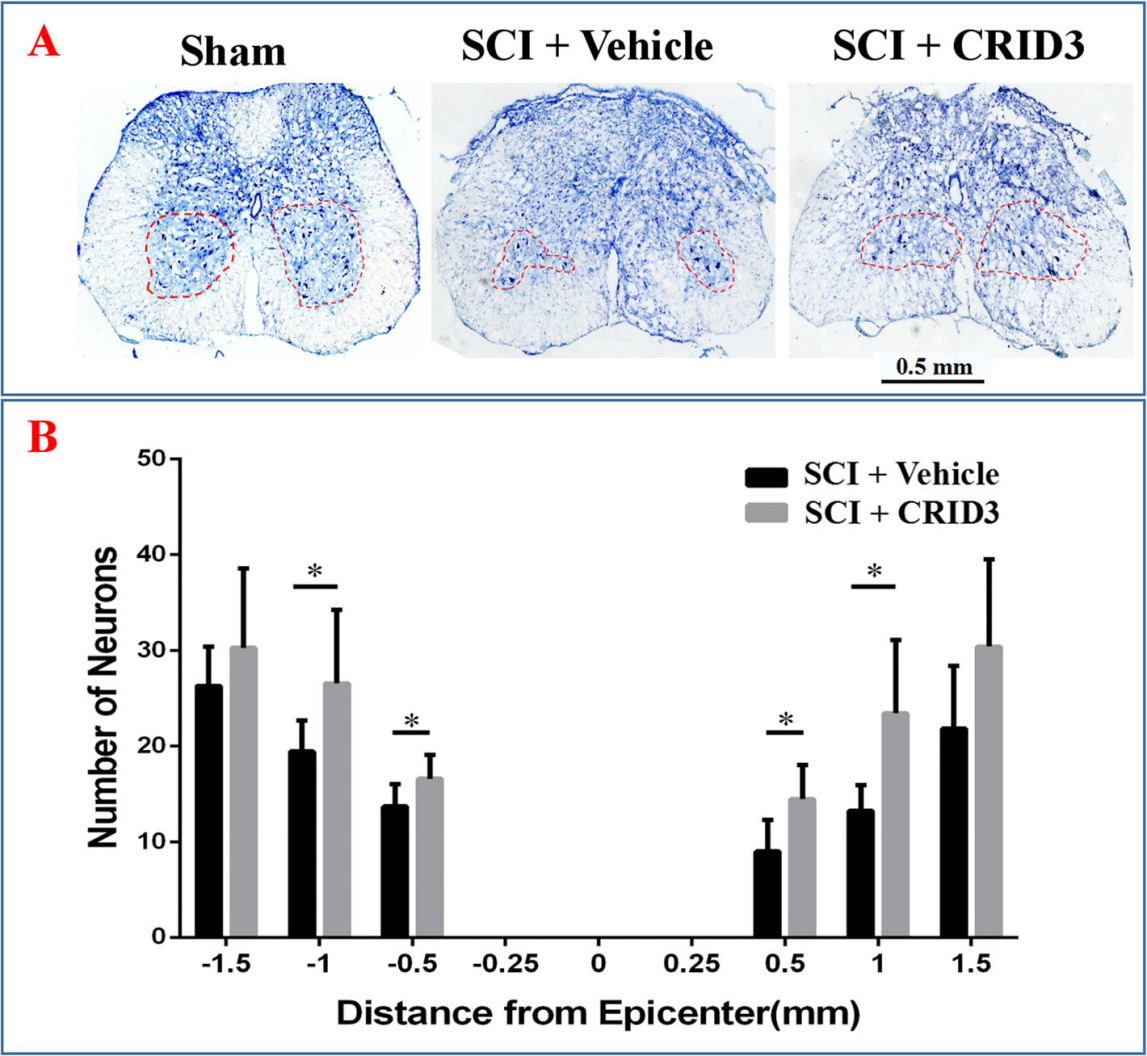
Quantitative analysis of the effect of CRID3 on motor neuron survival 6 weeks after SCI. **a** Nissl-stained spinal cord cross section from 0.5 mm rostral to the epicenter and the corresponding sections of sham-opened spinal cord. **b** Quantitative analysis of residual ventral horn motoneurons in different groups at various distances from the injury epicenter (0) and 0.25, 0.5, 1, and 1.5 mm rostral (+) and caudal (−) to the epicenter. Data represent the mean ± SD (*n* = 10). *P < 0.05 (repeated measures two-way ANOVA, followed by post hoc analysis for multiple comparisons)

To determine the effects of CRID3 on histopathology and behavior after SCI, the fibrosis area, myelin preservation, and neuronal survival were detected by hematoxylin-eosin (HE), Luxol fast blue (LFB), and Nissl staining, respectively. Figures 8a and 9a are the representative pictures of HE and LFB staining in the transverse sections of injured center of spinal cords and the corresponding sections of sham-opened spinal cords. Figure 8b showed that the fibrosis areas at the lesion epicenter, 0.25 mm rostral and caudal, and 0.5 mm rostral to the epicenter in SCI (CRID3) group were smaller than those of SCI (vehicle) group (*P* < 0.01 or 0.05, *n* = 10). Figure 9b showed that the LFB-positive areas at the lesion epicenter and 0.25 mm rostral and caudal to the epicenter of SCI (CRID3) group were larger than those of SCI (vehicle) group (*P* < 0.01 or 0.05, *n* = 10). Figure 10a is the representative picture of Nissl staining in the transverse sections at 1 mm rostral to the injured epicenter and the corresponding section of sham-opened spinal cord. Figure 10b showed that the numbers of residual ventral horn motoneurons at 0.5 and 1 mm rostral and caudal to the epicenter in SCI (CRID3) group were more than those of SCI (vehicle) group (*P* < 0.05, *n* = 10).

**Fig. 11:**
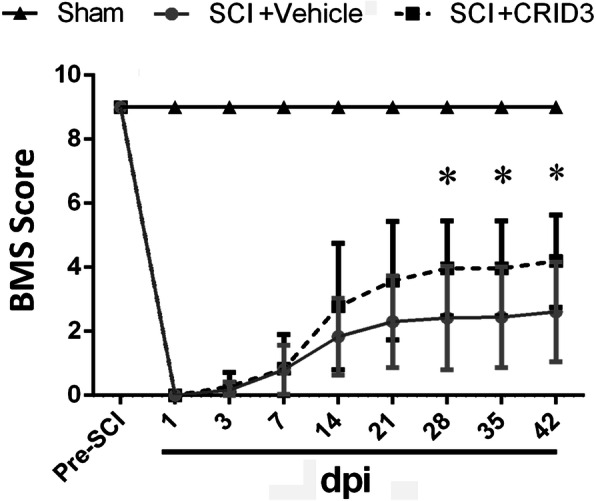
Effect of CRID3 on behavior recovery following SCI. Behavior recovery was evaluated by BMS. Data represent the mean ± SD (*n* = 10). *P < 0.05 (repeated measures two-way ANOVA, followed by post hoc analysis for multiple comparisons)

To investigate the effect of CRID3 on behavior recovery following SCI, the BMS scores were evaluated at 1 and 3 days, then 1, 2, 3, 4, 5-, and 6-weeks post-injury. As shown in Fig. 11, the scores of sham group and pre-injury were all 9 points, and there were no significant differences between SCI (CRID3) and SCI (vehicle) group from 1 day to 21 days post-injury (dpi). At 28, 35 and 42 dpi, the scores in SCI (CRID3) group were increased compared with SCI (vehicle) group. There were significant differences between the two groups (*P* < 0.05, *n* = 10).

## Discussion

Inflammasome is a multiprotein complex, which can regulate caspase-1 activation and promote the maturation and secretion of IL-1β and IL-18 in the process of natural immune defense [45, 46]. It can also regulate pyroptosis and induce cell death under inflammatory and stress conditions [45–48]. Inflammasomes have been reported to be involved in many diseases, including atherosclerosis [49], type II diabetes [50], Alzheimer’s disease [51], and autoimmune diseases [52]. Some inflammasomes, such as NLRP1, NLRP2, NLRP3, and AIM2, have been demonstrated play important roles in the inflammation of CNS injury [53, 54]. Although there are many different inflammasomes, ASC is their common adaptor protein [55]. Therefore, we speculate that ASC can be used as a target to inhibit the activation of inflammasomes, so as to improve the local immune microenvironment of SCI and reduce nerve damage. Previous studies have identified that CRID3 can inhibit ASC oligomerization in response to the stimulation of inflammasomes and further inhibit caspase-1 processing, IL-1β secretion, and pyroptosis in murine models of traumatic brain injury, dermal and pulmonary inflammation [18, 56, 57]. Our WB results also show that CRID3 can inhibit ASC expression and the activation of its related molecules, such as caspase-1, IL-1β, and IL-18, following SCI. Therefore, our study demonstrates that CRID3 can also affect the expression of inflammasome downstream inflammatory factors by inhibiting ASC oligomerization in the murine model of SCI.

Following SCI, inflammation is characterized by activated and increased inflammatory cells in the injured area, resulting in production of inflammatory factors, formation of inflammatory microenvironment, and eventually dysfunction [6]. Previous studies have shown that under natural conditions, the local immune microenvironment of SCI is “Yin-Yang” imbalance, and inflammatory cell subsets (such as Th1, Th17, Tc, and M1) and factors (such as IL-1β, IL-18, IL-6, IFN-γ, and TNF-α) are dominant, while anti-inflammatory cell subsets (such as Th2, Treg, Ts, and M2) and factors (such as IL-4, -10, -13, and TGF-β) are very rare [5, 6, 58–62]. This is an important mechanism of pathological damage following SCI. Since CRID3 can inhibit ASC and reduce the production of IL-1β and IL-18, we speculate that CRID3 can also improve the local immune microenvironment and play a neuroprotective role.

To clarify the effect of CRID3 on immune cell subsets, we used the strategy of combination of IHF and FCM. To distinguish among peripheral infiltrated leukocytes, infiltrated macrophages and local microglia, CD45, a general marker of leukocytes [30, 31], CD11b, the common marker of macrophages and microglia [32], CD68, a common marker of activated macrophages and/or microglia [33, 34], and M1- (CCR7) and M2- (Arg1) specific markers [33] were detected. The results indicate that the microglia in the sham-opened spinal cords are inherent, including both M1 and M2 subtypes and the proportion is balanced; however, the infiltrated macrophages are very rare. Following SCI, the infiltrated macrophages increase and mainly belong to M1 type, and most of the local microglia transform into M1 type. CRID3 has no significant influence on the number and differentiation of infiltrated macrophages. However, as for the phenotype transformation of local microglia, CRID3 can not only significantly reduce M1 cells but also increase M2 cells following SCI. So, we speculated that microglia-derived M1 cells might play more important roles than those derived from macrophages following SCI and microglia are more likely to be the target cells of CRID3. This is consistent with the recent reports, which demonstrated that inhibiting inflammasome activation can diminish M1 microglia and improve M2 microglia in vivo and in vitro [63–65]. However, its effect on other immune cell subsets still needs to be further explored.

To distinguish among different Th subsets, a general marker (CD4) [37], as well as Th1- (T-bet), Th2- (GATA3), and Th17- (RORγ-t) specific markers [38, 39] were detected by IHF. We found that the number of Th1, Th2, and Th17 cells increase significantly following SCI, CRID3 can reduce the number of Th1 cells; however, it has no influence on Th2 and Th17 cells. To analyze the proportion of Th cell subsets, FCM was used. In vitro, the classical FCM method to detect Th subsets is to activate T cells with stimulus, and then detect their general markers (CD3 and CD4) [66], as well as Th1- (INF-γ), Th2- (IL-4), and Th17- (IL-17) specific cytokines [67, 68]. However, in the injured spinal cords, it is impossible to apply the same method as in vitro. To solve this problem, we used a panel of surface markers which include CD3, CD4, CD183 (CXCR3), and CD196 (CXR6). Here, CD3 and CD4 are general markers of Th subsets [66], CD183 is the marker of Th1 cells and CD196 is the marker of Th17 cells [42]. Based on these markers, CD3^+^CD4^+^CD183^+^CD196^−^ cells are Th1, CD3^+^CD4^+^CD183^−^CD196^+^ cells are Th17 and CD3^+^CD4^+^CD183^−^CD196^−^ cells are Th2. In addition, a special group of CD3^+^CD4^+^CD183^+^CD196^+^ substyle is named Th1Th17. Th1Th17 is an interesting Th subset, which can produce both Th1 and Th17 cytokines, such as IFN-γ and IL-17 [42, 69]. Based on its cytokines, we speculate that it is a group of Th cells with stronger inflammation. Maybe it is because of the limitation of methodology that it is difficult to detect this group of cells only by immunohistochemistry. Here, combining with FCM, we found that Th1Th17 cell subset is dominant in Th cells in the injured spinal cord. CRID3 can reduce the number of Th1 cells and increase Th2 cells. In proportion, it also decreases Th1Th17 subset. The final results should be to significantly inhibit Th1 and Th1Th17 and promote Th2 cell differentiation. In addition, the numbers and proportions of Treg and Tc have also been detected. Although they both increase significantly following SCI, CRID3 has no significant effect on their numbers and proportions. This is consistent with the recent reports, which demonstrated that inflammasome activation can destroy Th1/Th2 balance, produce predominant Th1/Th17 inflammatory response, and inhibiting inflammasome activation can restore the Th1/Th2 balance [70–72].

Taken together, CRID3 can improve the local immune microenvironment by inhibiting inflammasome activation, which consequently suppress M1 microglia, Th1 and Th1Th17 differentiation, and increase M2 microglia and Th2 differentiation. These findings support that CRID3 might provide neuroprotection and improve behavior following SCI.

To determine whether CRID3 provides neuroprotection and improves functional recovery following SCI, morphological and functional assays were performed. A significant decrease in spinal cord fibrosis area accompanied by increasing myelinated white matter and residual ventral horn motoneurons, as well as corresponding locomotor improvements, is observed upon CRID3 treatment, as compared to animals receiving vehicle treatment, demonstrating that CRID3 treatment provides neuroprotection and promotes functional recovery following SCI.

## Conclusions

In conclusion, this study elucidates a mechanism by which CRID3 may ameliorate murine SCI by inhibiting inflammasome activation, reducing proinflammatory factor production, restoring immune cell subset balance, and improving local immune microenvironment, and early administration of CRID3 may be a promising therapeutic strategy for SCI.

## Supplementary information

**Additional file 1: Figure S1.** Effect of CRID3 on the numbers and proportions of Treg in the injured spinal cord. C) Representative images of CD4 (red) and FoxP3 (green) expression in the spinal cords in sham, SCI (vehicle) and SCI (CRID3) groups. Cells were counterstained with Hoechst 33342 (blue) to visualize nuclei. (D) Quantitative analysis of CD4^+^FoxP3^+^ cells in the indicated groups. Data represent the mean ± SD (n = 6). **P < 0.01 (non-parametric Kruskal-Wallis ANOVA, followed by individual Mann-Whitney U tests). (E) Representative images of FCM in the spinal cords in sham, SCI (vehicle) and SCI (CRID3) groups. In the FSC/SSC pseudocolor plot, the same size "region" of lymphocytes was set for each sample, and then analyzed the proportion of Treg subset in the “region” of CD3^+^CD4^+^ in CD25/CD127 pseudocolor plots. (F) Quantitative analysis of the indicated cells in the indicated groups. Data represent the mean ± SD (n = 6). P > 0.05 (non-parametric Kruskal-Wallis ANOVA, followed by individual Mann-Whitney U tests).

**Additional file 2: Figure S2.** Effect of CRID3 on the numbers and proportions of Tc in the injured spinal cord. (A-C) Representative images of CD8 (green) and CD28 (red) expression in the spinal cords in sham, SCI (vehicle) and SCI (CRID3) groups. Cells were counterstained with Hoechst 33342 (blue) to visualize nuclei. (D) Quantitative analysis of CD8^+^CD28^+^ cells in the indicated groups. Data represent the mean ± SD (n = 6). **P < 0.01 (non-parametric Kruskal-Wallis ANOVA, followed by individual Mann-Whitney U tests). (E) Representative images of FCM in the spinal cords in sham, SCI (vehicle) and SCI (CRID3) groups. In the FSC/SSC pseudocolor plot, the same size "region" of lymphocytes was set for each sample, and then analyzed the proportion of Tc subset in the “region” of CD3^+^CD8^+^ in CD8/CD28 pseudocolor plots. (F) Quantitative analysis of the indicated cells in the indicated groups. Data represent the mean ± SD (n = 6). P > 0.05 (non-parametric Kruskal-Wallis ANOVA, followed by individual Mann-Whitney U tests).

## Data Availability

The datasets used and/or analyzed during the current study are available from the corresponding author on reasonable request.

## Abbreviations

ASC: Apoptosis-associated speck-like protein containing a card
BMS: Basso mouse scale
CNS: Central nervous system
CRID3: A blocker of ASC oligomerization
DAMPs: Damage-associated molecular patterns
HE: Hematoxylin-eosin
IHF: Immunohistofluorescence
FCM: Flow cytometry
LFB: Luxol fast blue
PAMPs: Pathogen-associated molecular patterns
PFA: Paraformaldehyde
SCI: Spinal cord injury
WB: Western blot
