# Supplementary figures and images for: CRID3, a blocker of apoptosis associated speck like protein containing a card, ameliorates murine spinal cord injury by improving local immune microenvironment
Source: J Neuroinflammation. 2020 Aug 29;17:255. doi: 10.1186/s12974-020-01937-8 (PMC7456508; doi:10.1186/s12974-020-01937-8)

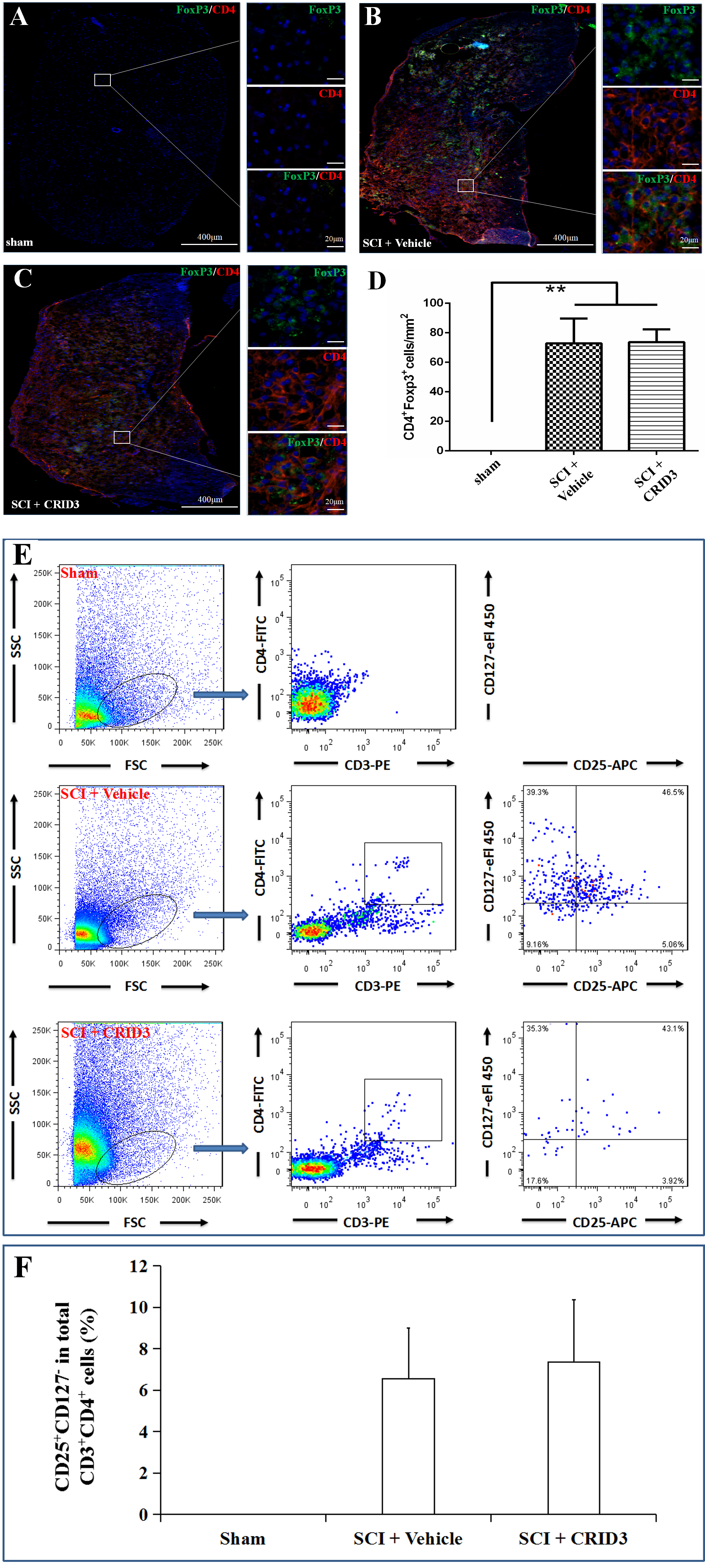

Supplement: Supplementary file 1 — Additional file 1: Figure S1. Effect of CRID3 on the numbers and proportions of Treg in the injured spinal cord. C) Representative images of CD4 (red) and FoxP3 (green) expression in the spinal cords in sham, SCI (vehicle) and SCI (CRID3) groups. Cells were counterstained with Hoechst 33342 (blue) to visualize nuclei. (D) Quantitative analysis of CD4+FoxP3+ cells in the indicated groups. Data represent the mean ± SD (n = 6). **P < 0.01 (non-parametric Kruskal-Wallis ANOVA, followed by individual Mann-Whitney U tests). (E) Representative images of FCM in the spinal cords in sham, SCI (vehicle) and SCI (CRID3) groups. In the FSC/SSC pseudocolor plot, the same size "region" of lymphocytes was set for each sample, and then analyzed the proportion of Treg subset in the “region” of CD3+CD4+ in CD25/CD127 pseudocolor plots. (F) Quantitative analysis of the indicated cells in the indicated groups. Data represent the mean ± SD (n = 6). P > 0.05 (non-parametric Kruskal-Wallis ANOVA, followed by individual Mann-Whitney U tests). [file 12974_2020_1937_MOESM1_ESM.tif]

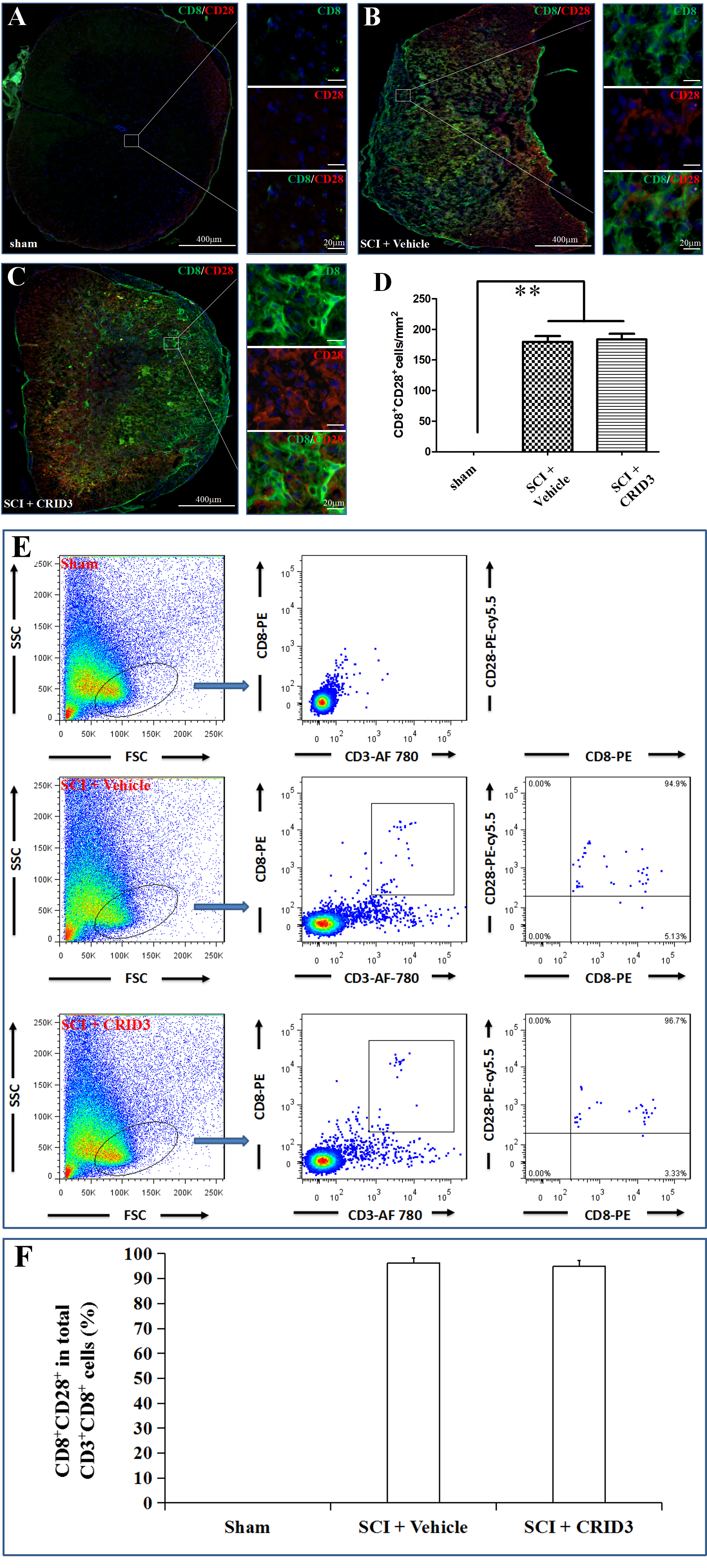

Supplement: Supplementary file 2 — Additional file 2: Figure S2. Effect of CRID3 on the numbers and proportions of Tc in the injured spinal cord. (A-C) Representative images of CD8 (green) and CD28 (red) expression in the spinal cords in sham, SCI (vehicle) and SCI (CRID3) groups. Cells were counterstained with Hoechst 33342 (blue) to visualize nuclei. (D) Quantitative analysis of CD8+CD28+ cells in the indicated groups. Data represent the mean ± SD (n = 6). **P < 0.01 (non-parametric Kruskal-Wallis ANOVA, followed by individual Mann-Whitney U tests). (E) Representative images of FCM in the spinal cords in sham, SCI (vehicle) and SCI (CRID3) groups. In the FSC/SSC pseudocolor plot, the same size "region" of lymphocytes was set for each sample, and then analyzed the proportion of Tc subset in the “region” of CD3+CD8+ in CD8/CD28 pseudocolor plots. (F) Quantitative analysis of the indicated cells in the indicated groups. Data represent the mean ± SD (n = 6). P > 0.05 (non-parametric Kruskal-Wallis ANOVA, followed by individual Mann-Whitney U tests). [file 12974_2020_1937_MOESM2_ESM.tif]
